# Growth on ATP Elicits a P-Stress Response in the Picoeukaryote *Micromonas pusilla*

**DOI:** 10.1371/journal.pone.0155158

**Published:** 2016-05-11

**Authors:** LeAnn P. Whitney, Michael W. Lomas

**Affiliations:** Bigelow Laboratory for Ocean Sciences, East Boothbay, Maine, United States of America; University Paris South, FRANCE

## Abstract

The surface waters of oligotrophic oceans have chronically low phosphate (P*i*) concentrations, which renders dissolved organic phosphorus (DOP) an important nutrient source. In the subtropical North Atlantic, cyanobacteria are often numerically dominant, but picoeukaryotes can dominate autotrophic biomass and productivity making them important contributors to the ocean carbon cycle. Despite their importance, little is known regarding the metabolic response of picoeukaryotes to changes in phosphorus (P) source and availability. To understand the molecular mechanisms that regulate P utilization in oligotrophic environments, we evaluated transcriptomes of the picoeukaryote *Micromonas pusilla* grown under P*i*-replete and -deficient conditions, with an additional investigation of growth on DOP in replete conditions. Genes that function in sulfolipid substitution and P*i* uptake increased in expression with P*i*-deficiency, suggesting cells were reallocating cellular P and increasing P acquisition capabilities. P*i*-deficient *M*. *pusilla* cells also increased alkaline phosphatase activity and reduced their cellular P content. Cells grown with DOP were able to maintain relatively high growth rates, however the transcriptomic response was more similar to the P*i*-deficient response than that seen in cells grown under P*i*-replete conditions. The results demonstrate that not all P sources are the same for growth; while *M*. *pusilla*, a model picoeukaryote, may grow well on DOP, the metabolic demand is greater than growth on P*i*. These findings provide insight into the cellular strategies which may be used to support growth in a stratified future ocean predicted to favor picoeukaryotes.

## Introduction

Picophytoplankton (< 3 μm), composed of both prokaryotic and eukaryotic organisms, dominate autotrophic biomass in oligotrophic oceans. While single-celled cyanobacteria are the most abundant autotrophs, picoeukaryotes can dominate biomass and productivity in the subtropics [1–3], making them important contributors to ocean carbon production [4] and export [5]. In the central North Atlantic Ocean, picoeukaryotes accounted for approximately 87% of the carbon biomass and 68% of the picophytoplankton primary production [1]. Furthermore, eukaryotes in the subtropical North Atlantic Ocean were found to be biochemically different than co-occuring prokaryotic lineages with a higher δ^15^N signature and were estimated to be responsible for nearly all of the new production [6].

Phosphorus (P) is an essential macronutrient utilized by phytoplankton for growth and, as such, has the potential to significantly influence oceanic primary production [7–11]. Oligotrophic oceans, like the North Atlantic subtropical gyre, have consistently low (<10 nmol L^-1^) phosphate (P*i*) concentrations during stratified periods [12, 13] although it can reach up to >20 nmol L-1 during periods of deep convective mixing [11]. In this region, dissolved organic phosphorus (DOP) is an important nutrient source accounting for >80% of the total dissolved P [8, 12, 13] and is readily utilized by the resident phytoplankton [11, 14–16]. Modeling studies have demonstrated that DOP may be supplied to the subtropical North Atlantic through horizontal transport from the northwest African shelf region [10, 17, 18] where there is a net DOP production [19]. As surface oceans warm and stratification increases, these cross-basin sources of organic nutrients may become progressively more important in supporting production in oligotrophic gyres.

Blooms of the pico-prasinophyte *Micromonas pusilla* have been observed in the subtropical North Atlantic with maxima in abundance associated with mixing events and high ambient DOP concentrations [11, 20]. *M*. *pusilla* has also been shown to be an important member of the picoeukaryote community in the Arctic [21]. Given its vast geographic range *M*. *pusilla* has been proposed as a sentinel organism for understanding the effects of climate change on biogeochemical cycling [22]. Despite its ecological importance the metabolic strategies employed by *M*. *pusilla* to cope with P*i*-deficiency and growth on different P sources are poorly understood. In general, marine phytoplankton elicit a three-pronged response to combat P stress which includes increasing P*i* uptake, reducing cellular P demand, and utilizing DOP. Indeed P-limited *M*. *pusilla* cultures have been shown to increase alkaline phosphatase activity (APA; [23]), reduce their cellular P quota [23], and adjust their lipid composition [24]. However, the molecular underpinnings driving these physiological responses remain unknown. Furthermore, the molecular and cellular response to growth on DOP has not been explored.

Here, transcriptomics were used to investigate the whole-genome expression response of *M*. *pusilla* to P scarcity and P source. RNA-sequencing along with cellular macronutrient composition, and alkaline phosphatase activity (APA) were used to characterize the cellular response to P*i*-replete and P*i*-deficient conditions. A significant increase in APA and expression of genes that function in P acquisition concurrent with a decrease in growth rate and cellular P content were expected in the P*i*-deficient cultures. Given the importance of DOP to picophytoplankton in oligotrophic oceans, we also investigated the response in *M*. *pusilla* cultures grown under replete conditions with ATP as the only P source. With the exception of increasing APA and the corresponding gene expression, we hypothesized growth rates and elemental composition to be similar in P*i*- and ATP-replete *M*. *pusilla* cultures due to growth in an equimolar P environment.

## Materials and Methods

### Culture conditions and physiological measurements

Duplicate (denoted ‘a’ and ‘b’), axenic batch *M*. *pusilla* (CCMP 2709) cultures (3 L) were grown under P*i*-replete, ATP-replete, and P*i*-deficient conditions at 16°C in a light:dark cycle of 14:10 h at 120 μE m^-2^ s^-1^. Prior to the start of the experiment, the *M*. *pusilla* culture was evaluated for the presence of bacteria by SYTO-staining and processing via flow cytometry [25], while throughout the experiment bacterial contamination was assessed using L1pm media [26]; all samples were negative. The experimental cultures were inoculated with exponentially growing, P*i*-replete *M*. *pusilla* cells that had been spun, washed, and finally resuspended in the treatment media at a starting concentration of ~ 1.6 x 10^5^ cells mL^-1^. Cultures were bubbled with 0.2 μm filtered 380 ppm compressed air:CO_2_ mix. All media, as well as the culture used for the inoculum, were equilibrated to the pCO_2_ condition prior to the start of the experiment. The pCO_2_ levels were controlled and monitored as the work presented here is part of continuing research aimed at understanding the impact of changing pCO_2_ on picoeukaryote growth.

Cells were grown in artificial sea water [22] amended with either autoclaved (macronutrients and trace-metals) or 0.2 μM syringe-filter sterilized (vitamins) L1 nutrients [26] with the exception of P and silicate, which were omitted. Phosphorus was added separately to achieve the desired condition; P*i*-replete media contained 36 μM PO_4_^3-^, the P*i*-deficient media received 0.5 μM PO_4_^3-^, and the ATP-replete treatment contained 12 μM ATP (~ 36 μM P). Cells grown in the P*i*-deficient treatment are expected to be limited at this concentration based on the scaling relationship between P*i* utilization and cell volume (e.g., [27]) which suggests a 2 μm cell would have a half-saturation concentration (K_m_) for P*i* uptake of ~0.1 μM; a similar K_m_ value for growth on P*i* was also reported in the prasinophyte *Prasinomonas capsulatus* [28]. Over half of the DOP pool is unidentified; ATP was selected as the proxy for DOP as it represents a compound that not only is quantifiable but also has been detected in every marine environment where measurements have been made [29].

Growth was monitored daily by fluorescence measurements using a Turner TD-700 Fluorometer (Sunnyvale, CA) and cell counts which were analyzed by flow cytometry. Samples for cell abundances were fixed with paraformaldehyde (0.5% final concentration), incubated for one hour at 4°C, and stored at -80°C until analysis. Samples were analyzed on a BD FACSJazz cell sorter (San Jose, CA); cells were enumerated and converted to cell abundances using the volume analyzed method [30]. Temperature and pH measurements were also made daily using an Orion Star A211 pH meter (Thermo Scientific, Waltham, MA).

**Fig 1:**
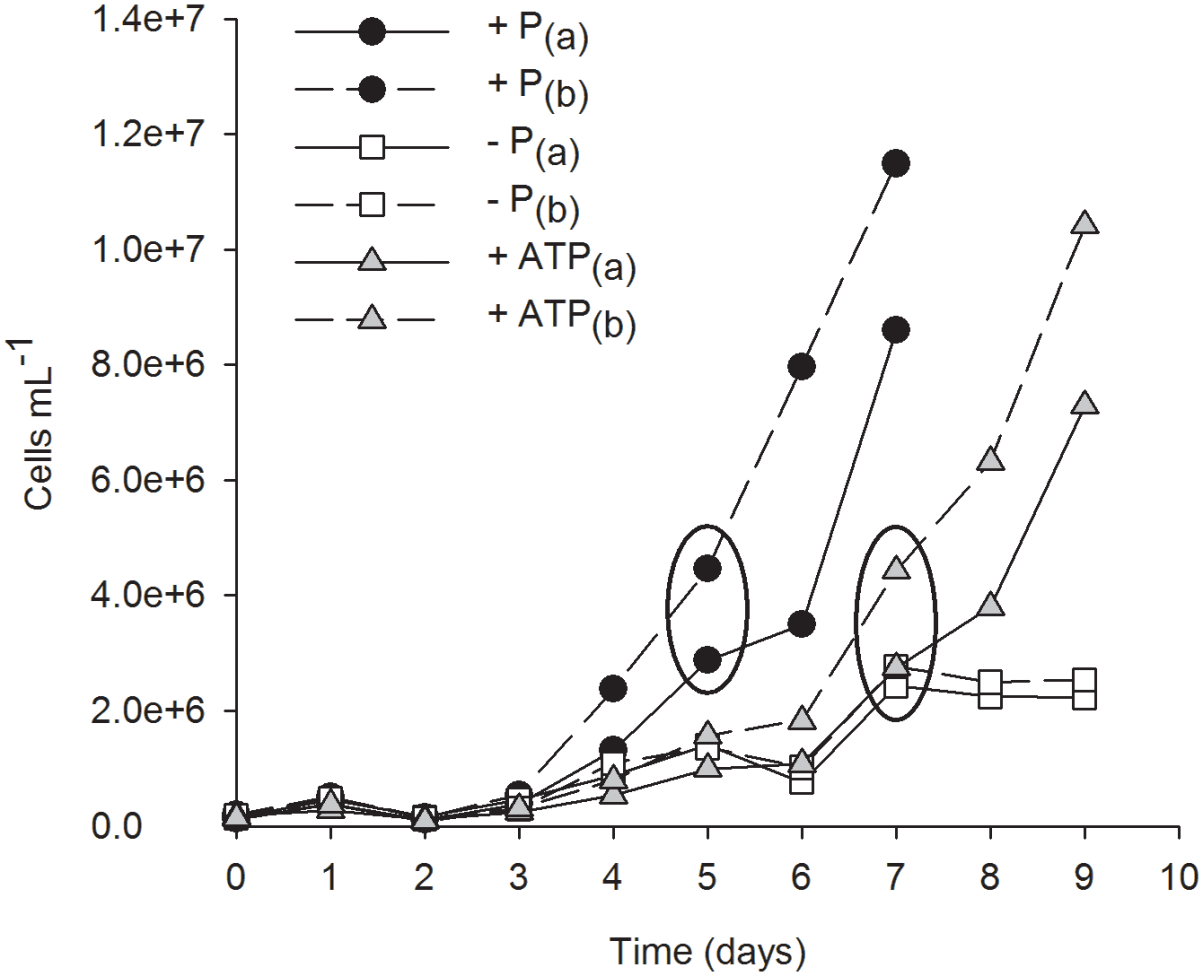
Growth curves for *Micromonas pusilla* under P*i*-replete (+ P), P*i*-deficient (- P), and ATP-replete (+ ATP) conditions. (a) and (b) represent culture replicates. Ellipses indicate time of harvest.

Samples were collected for dissolved and cellular nutrient analysis, APA, salinity, and total alkalinity (A_T_) at the beginning of the experiment (day 1) and on day 5 for the P*i*-replete treatment and day 7 for the P*i*-deficient and ATP-replete treatments. Cells from the P*i*-replete and ATP-replete cultures were harvested in early exponential phase and P*i*-deficient cells were harvested when growth was reduced when compared to the other treatments (Fig 1). The harvest times were selected so as to capture strong changes in gene expression associated with growth on different P sources, when cell abundances were high enough to support the desired analyses, as well as when pH (S1 Fig) and carbon chemistry changes due to cell growth and biomass accumulation were minimal (S1 Table). Nutrient samples were filtered through 0.2 μm polycarbonate filters and stored in HDPE bottles at -20°C until analyzed. Nitrate and phosphate concentrations were measured using a Seal AA3HR Segemented Flow Autoanalyzer (Mequon, WI). Samples for A_T_ were 0.2 μm filtered and stored in sealed glass vials until analyzed. Duplicate A_T_ measurements were made via titration using 0.1 N HCl and a Metrohm 888 Titrando (Herisau, Switzerland). Certified reference material [31] were included in the measurements. Culture pCO_2_ concentrations and DIC were calculated using CO2SYS Software [32] using constants from Mehrbach [33], refit by Dickson and Millero [34], and accounting for P*i* concentrations (S1 Table).

Cell samples for particulate carbon (C), nitrogen (N), and phosphorus (P) were collected onto precombusted 25 mm Whatman glass fiber filters (GE Healthcare Bio-Sciences, Pittsburgh, PA) and stored at -20°C. Particulate C and N samples were dried and analyzed on a Costech 4040 elemental analyzer (Valencia, CA) using acetanilide as a standard. Particulate P determinations were made as described by Lomas et al [11]. Briefly, filters were rinsed with 0.017 M MgSO_4_, dried at 90°C, and combusted at 500°C for 2 h. Upon cooling, 0.2 M HCl was added and hydrolyzed at 80°C for 30 min. After cooling, mixed reagent [35] was added, the samples were centrifuged, and absorbance was read at 885 nm using a Genesys 10 spectrophotometer (Thermo Scientific).

Triplicate APA measurements were made by quantifying the hydrolysis of 6,8-difluoro-4-methylumbelliferyl phosphate (Life Technologies, Grand Island, NY) using a Molecular Devices FilterMax F5 microplate reader (Sunnyvale, CA). Abiotic substrate hydrolysis was accounted for in killed controls that were boiled and cooled prior to substrate addition, as well as in media-only controls. The fluorescent reference standard, 6,8-difluoro-4-methylcoumarin (Life Technologies) was used to calculate the rate of hydrolysis, which was then normalized to cell abundance to determine APA per cell.

Additionally, daily APA measurements were made in a separate *M*. *pusilla* batch experiment. Growth conditions were similar to those previously described with the following exceptions: cultures (1.5 L) were grown in triplicate for each treatment and the starting cell density was greater (~ 5.5 x 10^5^ cells mL^-1^). Finally, two P*i*-deficient cultures received a P*i* addition (36 μM) on day 5 to demonstrate cells were indeed limited by P availability.

### Statistical Analysis

Analysis of variance (ANOVA) tests were conducted using SigmaStat (version 3.5; Systat Software, San Jose, CA) to determine statistically significant differences among APA measurements collected in the independent culture experiment where cultures were grown in triplicate for each treatment.

### RNA preparation and transcriptome sequencing

Approximately 1.5 L of culture volume was gently filtered over 0.8 μm, 47 mm polycarbonate filters on day 5 for the P*i*-replete and day 7 for the P*i*-deficient and ATP-replete treatments. Filters were stored in lysis buffer, flash frozen, and stored at -80°C until analyzed. Total RNA was extracted using the Qiagen RNeasy Mini Kit (Venlo, Netherlands) according to the manufacturer’s protocol, with the following exceptions: cells were lysed using 0.5 mm zirconia/silica beads (BioSpec, Bartlesville, OK, USA) mixed with the lysis buffer and vortexed until the solution appeared homogenous. The lysis solution was then passed through Qiashredder columns (Qiagen) to remove any large cell material that could clog the spin columns. To aid in the removal of DNA, two DNase digestions were performed. First, Qiagen’s RNase-free DNase Set (an on-column treatment) was used according to the manufacturer’s instructions. The second DNA removal step was conducted using the Turbo DNA-free kit (Life Technologies) according to the manufacturer’s protocol. The RNA was then quantified in duplicate using a Qubit Fluorometer (Life Technologies); RNA quality was assessed by gel electrophoresis.

RNA preparation and sequencing were performed by the U.S. Department of Energy Joint Genome Institute (JGI; sequencing project ID 1042280). RNA sequencing libraries were generated from 1 μg of RNA with 100 base pair paired-end reads sequenced using an Illumina HiSeq 2000. Reads were analyzed following the JGI pipeline. First, read quality was assessed using BBDuk [36] where artifact sequences were detected by kmer matching (kmer = 25) and trimmed. Reads were then quality trimmed using the phred trimming method at Q6 and finally, reads under 25 bases were removed. The remaining reads from each library were aligned to the *M*. *pusilla* genome [22] using TopHat [37] with only unique mapping allowed. Gene counts for each culture replicate were generated by featureCounts [38]; Pearson’s correlation (r) was used to demonstrate the high reproducibility among biological replicates within each treatment (r = 0.97, 0.94, 0.81 for P*i*-replete, P*i*-deficient, and ATP-replete, respectively). DESeq2 [39] was used to determine differential expression between the P*i*-replete and P*i*-deficient treatments as well as between the P*i*-replete and ATP-replete growth conditions. Differentially expressed genes are those with a p-value <0.05 and a fold change >2 (S2 Table).

Genes that were differentially expressed in at least one treatment were compared in a hierarchical cluster analysis using Cluster 3.0 [40]. Average counts were log transformed, centered about the mean, and normalized by multiplying each gene by a scale factor so that the sum of the squares of the values for each gene is 1. A centered correlation was used as a similarity metric for both the genes and treatments; a complete linkage was used as the clustering method. Java Tree View [41] was used to read and display the data.

## Results

### Cellular response to the transition of P*i*-replete growth to ATP-replete and P*i*-deficient growth conditions

The P*i*-replete and ATP-replete cultures received an equal amount of P, yet the growth rate of cells grown with ATP was ~30% less than that of P*i*-replete cells (Fig 1; Table 1). Cells from both treatments were harvested during the exponential phase of growth (Fig 1); at this time P*i* concentrations had stayed the same or increased in the ATP-replete cultures (Table 1). The growth rate of P*i*-deficient cells decreased by ~75% when compared to the P*i*-replete treatment (Table 1).

**Table 1:** *Micromonas pusilla* growth rates and culture media nutrient concentrations.

Treatment	Growth rate (d^-1^)^a^	NO_3_ (μM)^b^	PO_4_ (μM)^b^	Change in PO_4_ (μM)^c^
+ P_(a)_	0.84	810.3	28.5	-4.1
+ P_(b)_	0.87	818.2	28.6	-7.2
- P_(a)_	0.20	883.7	0.2	-0.2
- P_(b)_	0.21	846.6	0.1	-0.3
+ ATP_(a)_	0.61	890.8	0.4	0.0
+ ATP_(b)_	0.56	836.0	0.9	0.4

^a^ Days 2–7, 5–9, and 6–9 were used to calculate growth rates for the + P, − P, and + ATP cultures, respectively.

^b^ Nitrate and phosphate concentrations at the time of harvest.

^c^ Represents the difference in phosphate concentrations at the beginning of the experiment to the time of harvest.

A pronounced effect of changing the P availability was a decrease in the cellular P content of P*i*-deficient and ATP-replete cells (Table 2). Cellular P content decreased by approximately 50% and 25% in P*i*-deficient and ATP-replete cells, respectively, when compared to P*i*-replete cells (Table 2). P*i*-deficiency also resulted in increased cellular C content, while cellular N content was largely unaffected by the different treatments (Table 2). The increase in cellular C in P*i*-deficient cells could have been caused by an increase in cell size. Using forward scatter, determined by flow cytometry, as a proxy for size [2, 42], the P*i*-deficient cells were found to be slightly larger when compared to the P*i*-replete cells, although the increase in C content was ~20% (Table 2). These changes to the cellular elemental composition resulted in elevated C:P and N:P ratios in the P*i*-deficient and ATP-replete conditions (Table 2).

**Table 2:** *Micromonas pusilla* cellular carbon (Q_C_), nitrogen (Q_N_), and phosphorus (Q_P_) quotas.

Treatment	Q_C_ (fmol·cell^-1^)	Q_N_ (fmol·cell^-1^)	Q_P_ (fmol·cell^-1^)	C:N (mol:mol)	C:P (mol:mol)	N:P (mol:mol)	Relative cell size^a^
+ P_(a)_	134.2	19.6	1.2	6.8	114.2	16.7	1.00
+ P_(b)_	142.2	19.7	1.1	7.2	130.7	18.1	1.00
- P_(a)_	184.9	20.5	0.5	9.0	339.9	37.7	1.03
- P_(b)_	150.6	18.8	0.5	8.0	304.7	38.0	1.06
+ ATP_(a)_	143.0	24.0	0.8	6.0	176.3	29.6	0.98
+ ATP_(b)_	148.4	18.2	0.8	8.1	193.5	23.8	0.98

^a^ Ratio of P*i*-deficient and ATP-replete cell size when compared to the P*i*-replete cells.

**Fig 2:**
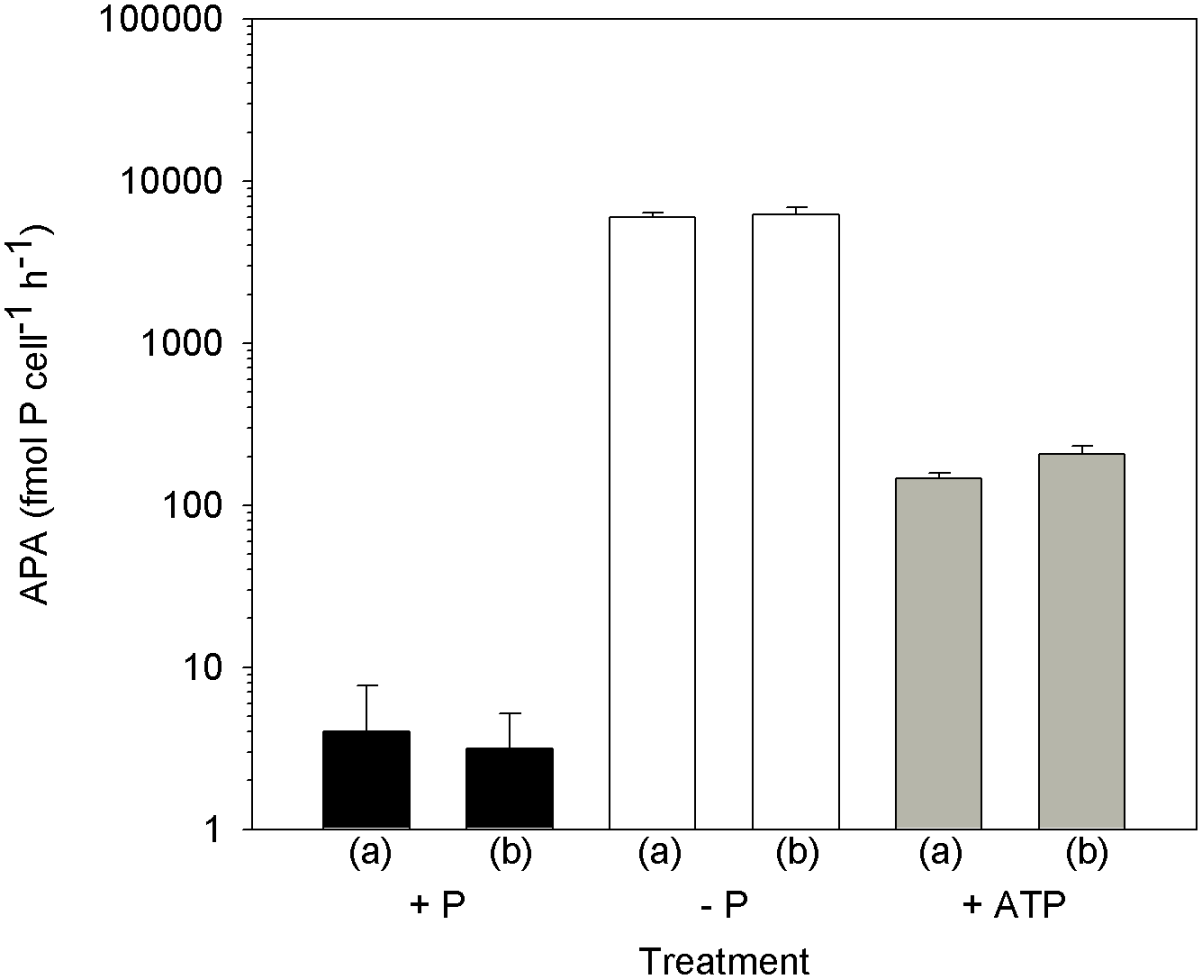
*Micromonas pusilla* alkaline phosphatase activity (APA) at time of harvest. (a) and (b) represent culture replicates. Error bars represent the standard deviation of triplicate measurements of each culture replicate.

**Fig 3:**
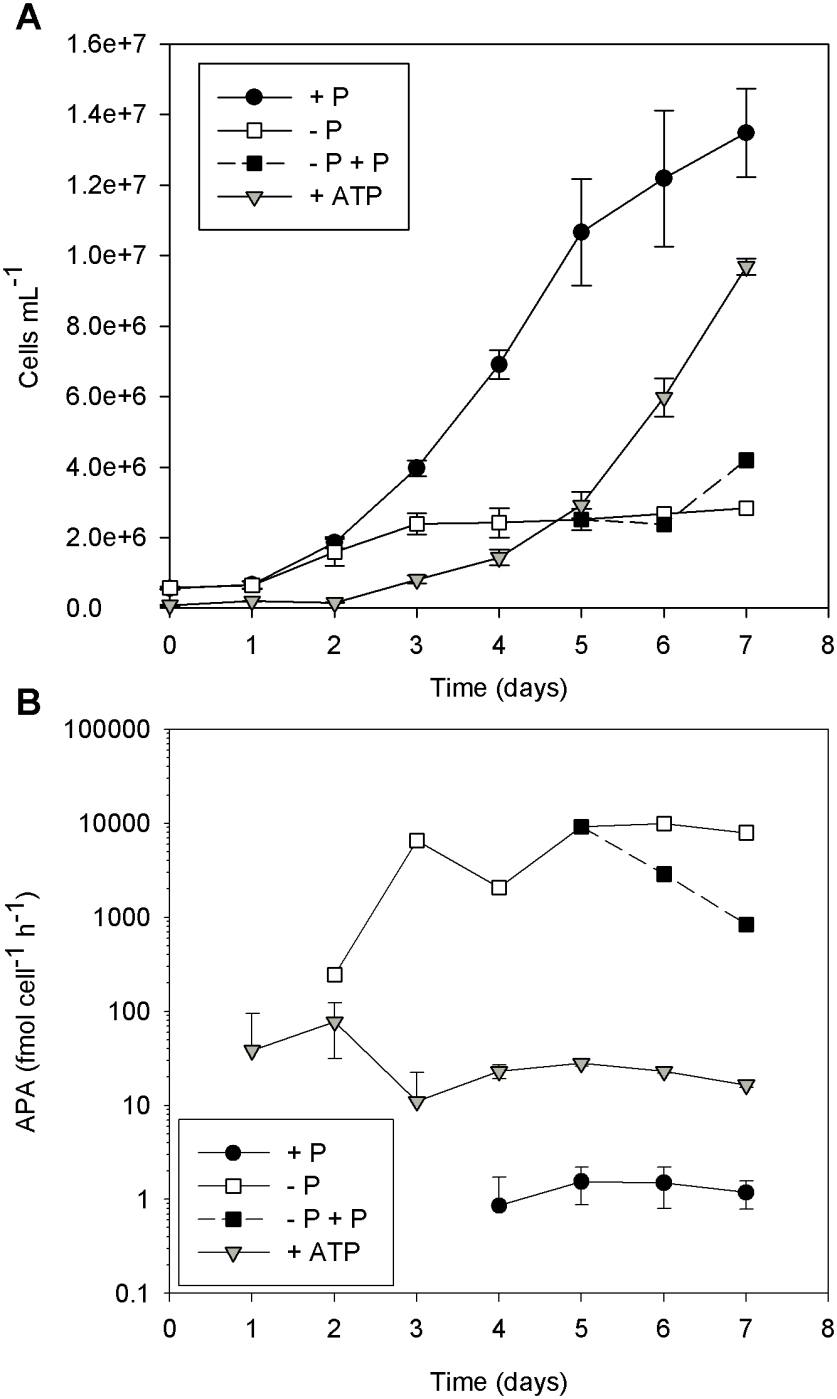
*Micromonas pusilla* cell abundance (A) and alkaline phosphatase activity (APA; B) when grown under P*i*-replete (+ P), P*i*-deficient, and ATP-replete (+ ATP) conditions. Cultures were grown in triplicate for each treatment. APA measurements began on day 1; missing data points indicate APA was not detected. On day 5, two of the P*i*-deficient cultures received P*i* additions (36 μM; − P + P). Error bars represent the standard deviation of cell abundance and APA measurements for the culture replicates for each treatment.

The highest APA levels were measured in the P*i*-deficient cultures with rates significantly greater than both P*i*-replete and ATP-replete cultures (Fig 2; p < 0.05). APA measurements were made when cell abundances of the P*i*-deficient cultures deviated from the ATP-replete cultures (Fig 1). In a separate growth experiment (Fig 3A), APA was measured daily in cultures where a deviation in growth was detected earlier (Fig 3B). In that experiment, APA levels in the ATP-replete and P*i*-deficient cultures remained relatively stable on days 6 and 7 (Fig 3B), demonstrating a difference in response among the treatments.

### Differential expression due to P*i*-deficiency and transition to DOP-replete growth

**Fig 4:**
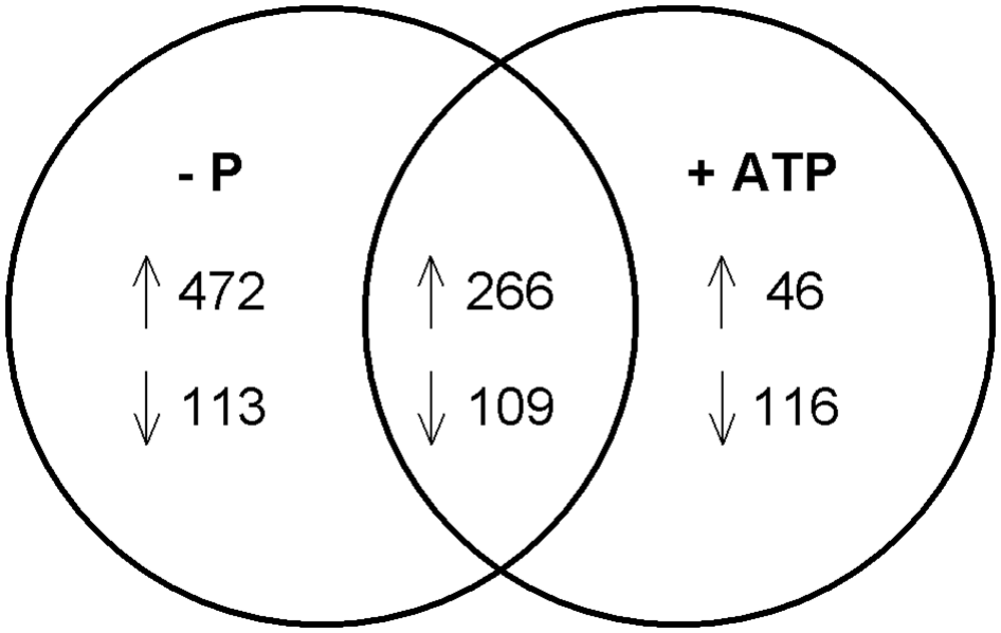
Venn diagram showing numbers of differentially (p < 0.05) expressed genes in P*i*-deficient and ATP-replete cells when compared to P*i*-replete. Overlapping section represents transcripts that are shared between the treatments. Up and down arrows indicate expression increased or decreased, respectively.

RNA-sequencing was used to characterize whole-genome expression patterns in P*i*-replete, P*i*-deficient, and ATP-replete *M*. *pusilla* cultures. Over 30 million sequence reads were generated for each culture with approximately 40% mapping to the *M*. *pusilla* genome [22], detecting nearly every protein-coding gene (Table 3). DESeq2 [39] was used to determine which genes were differentially expressed (p < 0.05) in the P*i*-deficient and ATP-replete treatments relative to the P*i*-replete condition. The differential transcriptomic response was greatest in the P*i*-deficient treatment with 960 differentially expressed genes compared to 537 in the ATP-replete condition (Fig 4). There were many differentially expressed genes shared between the two treatments (Fig 4), indicating ATP-replete cells responded similarly to the P*i*-deficient cells.

**Table 3:** Summary statistics of the *Micromonas pusilla* transcriptomes. The values represent the average of sequencing the duplicate cultures for each treatment.

Treatment	Total reads	% mapped	% genes detected
+ P	31,728,942	40	98
- P	31,552,587	40	98
+ ATP	32,889,716	40	98

**Fig 5:**
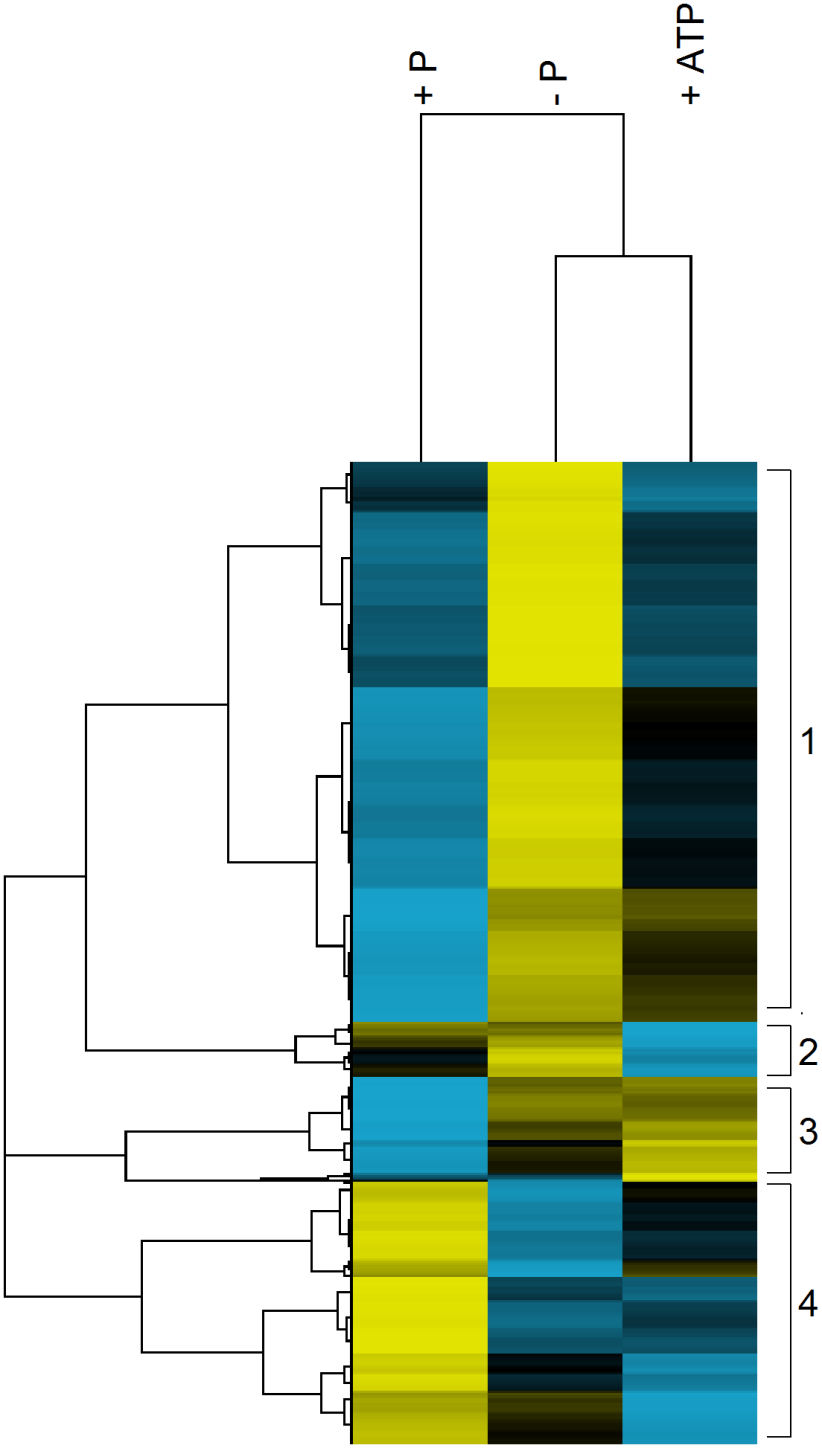
Hierarchical cluster analysis of the differentially expressed transcripts. Transcripts are grouped by similarity of patterns. The read counts for each transcript were averaged across treatments. Yellow indicates higher abundance than the mean while blue indicates reduced abundance relative to the mean. Black indicates no difference from the mean. The intensity of the color is indicative of the degree of difference from the mean, with brighter colors displaying stronger differences. Numbers indicate clusters of similar pattern.

**Fig 6:**
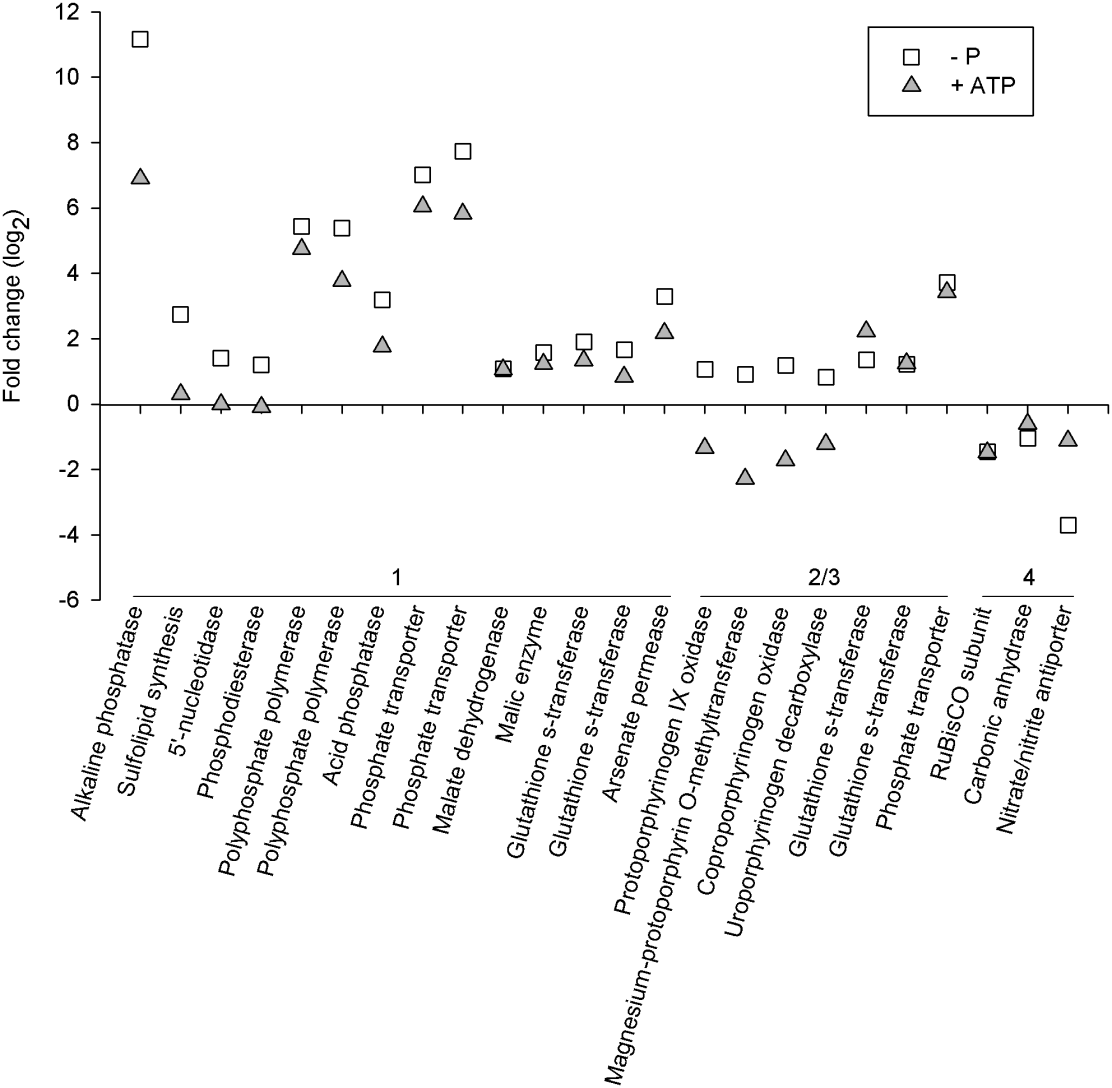
Expression patterns of P stress-response genes in P*i*-deficient and ATP-replete *Micromonas pusilla* cells when compared to P*i*-replete. Numbers refer to the cluster as shown in Fig 5.

A hierarchical cluster analysis [40] was performed to group the differentially expressed genes by similar expression patterns (Fig 5). The P*i*-replete treatment clustered separately indicating the ATP-replete and P*i*-deficient transcriptomes were more similar to each other than to P*i*-replete (Fig 5). Four clusters, or expression patterns, were generated. Cluster 1 contains transcripts that were repressed in P*i*-replete *M*. *pusilla* cells and so were over-represented in the P*i*-deficient and to a lesser extent, the ATP-replete treatments. Included in this cluster were P-stress response genes like AP (protein ID 64401), which was the most differentially expressed gene in both the P*i*-deficient and ATP-replete transcriptomes (Fig 6). P*i*-deficient cells also significantly upregulated transcripts encoding a sulfolipid synthase (protein ID 58169), 5’-nucleotidase (protein ID 106294), and a phosphodiesterase (protein ID 93904); these genes were not differentially expressed in the ATP-replete treatment (Fig 6). Also highly expressed in both P*i*-deficient and ATP-replete conditions were transcripts encoding genes involved in polyphosphate accumulation (protein ID 61436, 60787) and P*i* transporters (protein ID 108777, 84293; Fig 6). The transcriptomes indicated P*i*-deficient and ATP-replete *M*. *pusilla* cells were combating arsenic toxicity as several glutathione s-transferases (protein ID 57469, 63522) and an arsenate permease (protein ID 56091) were upregulated (Fig 6). Also found in Cluster 1 were several genes that may be involved in glycolytic bypass reactions. Transcripts encoding malate dehydrogenase (protein ID 75917) and malic enzyme (protein ID 97726) were upregulated in both P*i*-deficient and ATP-replete cells (Fig 6). Additionally, an acid phosphatase (protein ID 85046) was differentially expressed in the P*i*-deficient and ATP-replete treatments (Fig 6).

Clusters 2 and 3 contain transcripts reduced and over-represented in cells grown with ATP, respectively. Several genes involved in chlorophyll biosynthesis, including a protoporphyrinogen IX oxidase (protein ID 60613), magnesium-protoporphyrin O-methyltransferase (protein ID 96236), coproporphyrinogen oxidase (protein ID 104790), and an uroporphyrinogen decarboxylase (protein ID 104963), were significantly repressed when compared to the P*i*-replete treatment (Fig 6). Cluster 3 contains genes that were similar to those in Cluster 1 including transcripts encoding glutathione s-transferases (protein ID 73823, 107846; Fig 6) and a P*i* transporter (protein ID 53790; Fig 6).

The transcripts in Cluster 4 were found to accumulate in the P*i*-replete treatment (Fig 5). Genes involved in posttranslational modification, energy production, and carbon fixation were more abundant in P*i*-replete cells (Fig 6). For example, a RuBisCO subunit (protein ID 104787) as well as a carbonic anhydrase (protein ID 96952) had accumulated in the P*i*-replete *M*. *pusilla* cells. Additionally, transcripts for a nitrate/nitrite antiporter (protein ID 63387) were more abundant under P*i*-replete conditions (Fig 6).

## Discussion

Picoeukaryotes, though not numerically dominant, are equal to or may even exceed cyanobacteria in biomass, productivity, and export in the oligotrophic subtropical North Atlantic, where P stress is an important ecological determinant [1–3, 11]. Despite their role in ecosystem functioning, the cellular responses and molecular underpinnings to changes in P availability and supply are not well understood in picoeukaryotes. *M*. *pusilla* is considered a model organism [43] yet it has been the target of only two other studies which have interrogated changes in gene expression [44, 45]. Coupling molecular biology with model organisms provides insight into the interactions of phytoplankton with their environment [43]. Furthermore, characterizing phytoplankton physiological traits and capabilities is important for understanding how community structure may change in a changing marine environment [46]. We have chosen to characterize the response of *M*. *pusilla* to P scarcity and P source using batch culturing and transcriptomics. The P concentrations used in this study, though they do not represent what is found naturally in the oligotrophic North Atlantic, recreate the impact of low P availability by reducing growth rate while generating the biomass needed to support the desired analyses. This study is timely as it provides insight into the cellular metabolism of an ecologically important phytoplankton found in oligotrophic oceans which are predicted to expand [47] and become increasingly stratified [48].

### *Micromonas* elicits an extensive cellular response to P*i*-deficiency

In response to P*i*-deficiency, phytoplankton have been shown to reduce and reallocate their cellular P e.g., [49]), utilize DOP (e.g., [16]), and increase P uptake [50]. *M*. *pusilla* employs all of these strategies under P*i*-deficiency. As has been previously shown [23], the cellular P quota of P*i*-deficient *M*. *pusilla* cells was dramatically reduced when compared to P*i*-replete cells. Phytoplankton can reduce their P content by phospholipid substitution [49]. The upregulation of a sulfolipid biosynthesis gene suggests *M*. *pusilla* decreased its cellular P quota by swapping sulfolipids for phospholipids. Sulfolipid substitution has been detected in P*i*-deficient *M*. *pusilla* [24], diatom [51] and pelagophyte [52] cultures as well as naturally P-limited phytoplankton communities [49], indicating it is an important strategy to combat P stress. Differential expression of sulfolipid biosynthesis genes has also been detected in light-limited *Aureococcus anophagefferens* where it was likely responding to an increase in plastid membrane surface area and thus an increase in P demand [53]. Together, these results highlight the important role of sulfolipid swapping in maintaining a flexible P pool to support phytoplankton growth during suboptimal growth conditions.

Cellular P may also be conserved by inducing glycolytic bypass pathways. Under P deprivation, plants have been shown to generate pryruvate from phosphoenolpyrvate through the activity of phosphoenolpyruvate carboxylase resulting in oxaloacetate and a P*i* molecule [54]. Oxaloacetate is then converted to malate and finally pyruvate by malate dehydrogenase and malic enzyme, respectively [54]. Differential expression of phosphoenolpyruvate carboxlyase was not detected; however, the accumulation of malate dehydrogenase and malic enzyme transcripts suggests phosphoenolpyruvate may be diverted through this bypass. The induction of glycolytic bypass pathways under P*i*-deficiency has been seen in the diatom *Thalassiosira pseudonana* [51] as well as in several *Aureococcus anophagefferens* strains [52, 53] suggesting it may be a common strategy used by eukaryotic phytoplankton to combat P stress.

P*i*-deficient *M*. *pusilla* cells had high rates of APA. The induction of APA is a common strategy used widely among phytoplankton in response to P-stress [55]. Concurrent with the high level of APA was the accumulation of AP transcripts indicating P*i*-deficient *M*. *pusilla* cells are primed to acquire P from extracellular DOP sources. An acid phosphatase was also upregulated in P*i*-deficient cells; acid phosphatases catalyze the hydrolysis of P*i* molecules under acidic conditions. Acid phosphatase activity has been shown to increase in P-limited green algae where it may function in intracellular P recycling [56]. The acid phosphatase contains a signal peptide SignalP 4.1; [57]) suggesting it may be secreted. Perhaps the acid phosphatase is secreted to a polyphosphate vacuole where it could function in polyphosphate degradation. Polyphosphate is a linear polymer of P*i* molecules of variable length; cells can have multiple polyphosphate pools with different functions and regulation patterns [58]. Recent studies in phytoplankton reflect this complex modulation as P*i*-deficient cells have been shown to increase cellular polyphosphate [59, 60] or accumulate putative polyphosphate synthesis transcripts [52, 53, 59, 61]. Here, polyphosphate polymerase transcripts accumulated in P*i*-deficient *M*. *pusilla* suggesting cells were synthesizing polyphosphate in addition to utilizing it as a P source. P*i*-deficient *M*. *pusilla* cells may be using acid phosphatase to mobilize P from luxury uptake polyphosphate pools to generate P scavenging proteins or support key metabolic pathways, like photosynthesis.

Further evidence for the utilization of organic P is the upregulation of genes encoding 5’-nucleotidase and glycerophosphoryl diester phosphodiesterase. The lack of signal peptides suggests these enzymes function in intracellular P recycling. The 5’-nucleotidase hydrolyzes P*i* from nucleotides and has been shown to be induced in other eukaryotic phytoplankton under P*i*-deficient conditions [51, 62]. The induction of a gene encoding for a glycerophosphoryl diester phosphodiesterase indicates phospholipids may be recycled and used to sustain growth under P*i*-deficient conditions as has been shown in the diatom *T*. *pseudonana* [60]. The ability to utilize phosphodiesters as a P source is not ubiquitous among eukaryotic phytoplankton [63] or cyanobacteria [64]. Interestingly this *M*. *pusilla* strain was isolated from the South Pacific, a region well documented to have low P*i* [65]; this suggests an ecological advantage may be conferred to those that produce the enzyme when P*i* concentrations are at growth limiting levels.

Several P*i* transporters were strongly upregulated, suggesting P*i*-deficient cells were increasing P uptake. This is a strategy routinely used among phytoplankton to combat P stress (e.g., [50]). A PHO4-containing P*i* transporter (protein ID 61702) was identified in the *M*. *pusilla* genome suggesting it could be a high-affinity transporter as it is homologous to the high-affinity transporter gene identified in the prasinophyte *Tetraselmis chui* [66]. However, significant differential expression was not detected as transcript counts were either very low (P*i*-deficient and ATP-replete treatments) or zero (P*i*-replete). This could be indicative of a very stable protein that doesn’t require high transcript copy numbers. If so, *M*. *pusilla* could enhance P uptake not only by making more, but also by synthesizing high-affinity P transporters.

Phosphate transporters are unable to discriminate between P*i* and its analog, arsenic, making P-stressed cells susceptible to arsenic toxicity [67]. Detoxification strategies commonly include the reduction of arsenate to arsenite by arsenate reductase followed by its excretion out of the cell via an arsenite pump [68]. Glutathione s-transferases have also been shown to play an important role in alleviating arsenic stress in yeast [69] with recent evidence implicating its use by P*i*-deficient phytoplankton [53]. In the current study, several glutathione s-transferases were induced along with an aresenite permease suggesting cells have efficient arsenic detoxification strategies. The induction of arsenic detoxification genes under P-limiting conditions is not widespread among phytoplankton [51, 61] and could be indicative of the environment the phytoplankton commonly reside, like oligotrophic oceans where P*i* concentrations are chronically low.

### ATP elicits a muted P-stress response in *Micromonas*

The ATP-replete cultures maintained relatively high growth rates of 0.6 d^-1^ and reached similar cell abundances as the P*i*-replete cultures indicating *M*. *pusilla* was able to grow using ATP as a P source. To do this, *M*. *pusilla* elicited a cellular response that was similar to that seen in the P*i*-deficient treatment. Cells grown with ATP had reduced cellular P levels elevated APA, but not to the same extent as P*i*-deficient cells. A similar trend was observed in the transcriptome responses. Fewer genes were differentially expressed in the ATP-replete treatment when compared to P*i*-deficient cells and those that were sensitive were responding in both the P*i*-deficient and ATP-replete treatments.

The cluster analysis revealed that transcriptome expression patterns were similar between the ATP-replete and P*i*-deficient treatments. ATP-replete cells induced AP, polyphosphate polymerase, P transporters and arensic detoxification genes. The putative glycolytic bypass genes, malate dehydrogenase and malic enzyme, were also induced. Differential expression was not detected in genes that function in sulfolipid synthesis or recycling intracellular P via 5’-nucleotidase and phosphodiesterase. Taken together, these transcriptional changes suggest *M*. *pusilla* may be sensitive to the severity of P stress. Cells can regulate gene expression to balance P needed to support growth versus survival. If P*i* concentrations are low, cells reduce and recycle cellular P in addition to induce extracellular acquisition strategies. If DOP is present, a scaled back response is invoked which enables cells to acquire the P necessary to support cell growth and functioning.

Unique to the ATP-replete treatment was the decrease in chlorophyll biosynthesis gene expression. Nitrogen-deprivation has been shown to elicit a similar response in the diatom, *Phaeodactylum tricornutum* [70]. This, coupled with the accumulation of a nitrate/nitrite antiporter in P*i*-replete cultures, could indicate cells were nitrogen deficient, however the cellular N levels were similar among the treatments. Perhaps the ATP-replete cells reduced chlorophyll biosynthesis as a means to divert resources to produce proteins that function in DOP utilization. Differential expression of the chlorophyll biosynthesis genes was not detected with P*i*-deficiency. Here, *M*. *pusilla* may be conserving the already reduced photosynthetic and energy production capabilities [71] to generate the resources needed for producing P stress-response proteins. This hypothesis aligns with the notion that cells have a varied response that is sensitive to the severity of P stress.

### Ecological implications

The ecological importance of picoeukaryotes in P deplete oligotrophic oceans has only recently been recognized. We have provided insight into the strategies utilized by the picoeukaryote, *M*. *pusilla*, to persist in these suboptimal growth conditions. *M*. *pusilla* exhibited an extensive response to P*i*-deficiency that included efforts to acquire extracellular P, recycle intracellular P, as well as reduce their cellular P demand. This whole-cell metabolic reconfiguration may be necessary to maintain a foothold in oligotrophic oceans dominated by cyanobacteria that are extremely efficient at acquiring and growing at low P*i* concentrations [16]. *M*. *pusilla* is able to utilize alternative P sources like ATP to support growth, which is also essential for persisting in oligotrophic oceans [11]. Future oligotrophic oceans that are predicted to become increasingly stratified [48] and are slowly acidifying [72] could portend an enhanced role for picoeukaryotes. The abundance of *Micromonas*-like cells have been shown to increase in response to ocean acidification [73] while the cyanobacteria *Prochlorococcus* and *Synechococcus* are largely unaffected by elevated CO_2_ [74]. Additionally, elevated CO_2_ coupled with P-limitation promoted *M*. *pusilla* growth [23]. These results, combined with the strong, inducible response to P deficiency and their ability to efficiently grow using DOP shown in this study, support the hypothesis that future oceans could favor picoeukaryote growth [23]. Given their relatively larger cell size when compared to single-cell cyanobacteria and contribution to primary production [4] and export [5], this could have pronounced effects on the biogeochemistry of the oligotrophic oceans.

## Supporting Information

S1 Fig pH curves for *Micromonas pusilla* under P*i*-replete (+ P), P*i*-deficient (- P), and ATP-replete (+ ATP) conditions. (a) and (b) represent culture replicates. Data shown from day 1 to time of harvest.
(TIF)

S1 Table Culture media carbon chemistry at the time of harvest. (DOCX)

S2 Table Differentially expressed genes in P*i*-deficient and ATP-replete cultures when compared to the P*i*-replete treatment. (XLSX)
