# Supplementary figures and images for: Growth on ATP Elicits a P-Stress Response in the Picoeukaryote Micromonas pusilla
Source: PLoS One. 2016 May 11;11(5):e0155158. doi: 10.1371/journal.pone.0155158 (PMC4864187; doi:10.1371/journal.pone.0155158)

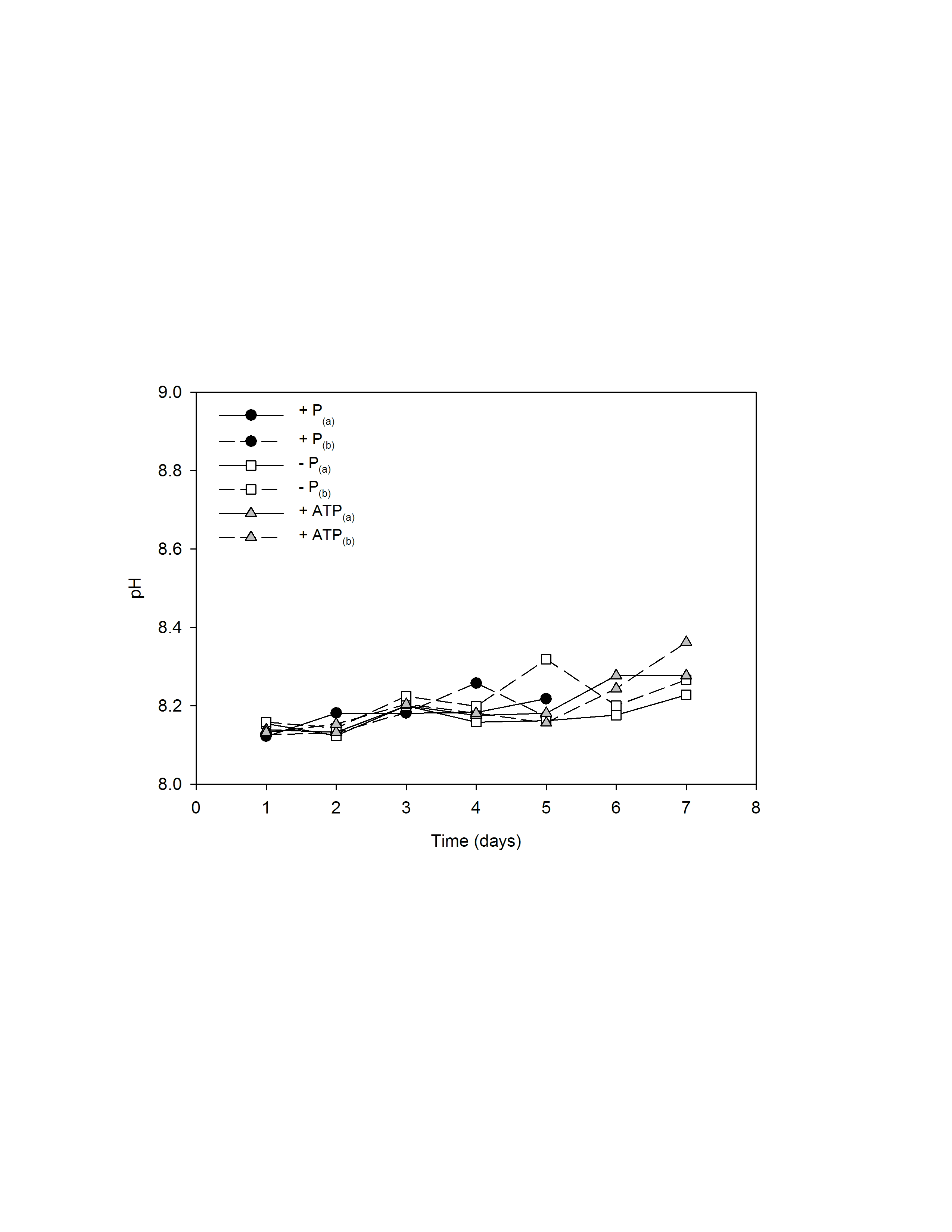

Supplement: S1 Fig — (a) and (b) represent culture replicates. Data shown from day 1 to time of harvest. (TIF) [file pone.0155158.s001.TIF]
