# Supplementary material for: Growth on ATP Elicits a P-Stress Response in the Picoeukaryote Micromonas pusilla
Source: PLoS One. 2016 May 11;11(5):e0155158. doi: 10.1371/journal.pone.0155158 (PMC4864187; doi:10.1371/journal.pone.0155158)
Supplement: S1 Table — (DOCX) [file pone.0155158.s002.docx]

| Table S1. Culture media carbon chemistry at the time of harvest. | | | | |
| --- | --- | --- | --- | --- |
|  |  |  |  |  |
| Treatment | pH | A_T_ (μEq kg^-1^) | pCO_2_ (μatm) | DIC (µmol kg^-1^) |
| + P_(a)_ | 8.22 | 2607.4 | 284 | 2240 |
| + P_(b)_ | 8.17 | 2648.9 | 328 | 2306 |
| - P_(a)_ | 8.23 | 2509.3 | 269 | 2172 |
| - P_(b)_ | 8.27 | 2597.9 | 250 | 2225 |
| + ATP_(a)_ | 8.28 | 2559.7 | 239 | 2185 |
| + ATP_(b)_ | 8.36 | 2586.2 | 188 | 2145 |
